# Evaluation of Ganglion Cell-Inner Plexiform Layer Thickness after Vitreoretinal Surgery with Internal Limiting Membrane Peeling in Cases with Idiopathic Macular Hole

**DOI:** 10.4274/tjo.34545

**Published:** 2017-06-01

**Authors:** Sibel Demirel, Ahmed Abdullayev, Özge Yanık, Figen Batıoğlu, Emin Özmert

**Affiliations:** 1 Ankara University Faculty of Medicine, Department of Ophthalmology, Ankara, Turkey

**Keywords:** Dissociated optic nerve fiber layer, ganglion cell-inner plexiform layer, Macular hole

## Abstract

**Objectives:**

To evaluate macular retinal ganglion cell-inner plexiform layer (GCIPL) thickness after vitrectomy with internal limiting membrane (ILM) peeling for idiopathic macular holes using spectral domain optical coherence tomography (SD-OCT).

**Materials and Methods:**

Eighteen eyes of 18 patients with unilateral idiopathic macular hole who underwent vitrectomy with ILM peeling were retrospectively analyzed. Healthy fellow eyes of the patients and 18 eyes of 18 age-matched healthy individuals constituted the control group. The patients were evaluated at 1 day, 1 week, 1 month, and 3 months after surgery. The best corrected visual acuity (BCVA) measurements, biomicroscopic examination findings and SD-OCT measurements were recorded. Ganglion cell-inner plexiform layer thickness was evaluated with ganglion cell analysis software of Cirrus HD-OCT before surgery and at 1 month and 3 months after surgery and compared with control groups. Presence of dissociated optic nerve fiber layer (DONFL) was evaluated with C-scan mode.

**Results:**

Of the 18 patients, 9 were male and 9 were female with a mean age of 65.6±5.6 (55-77) years. Preoperative BCVA was 0.75±0.19 logMAR, while it was 0.44±0.17 logMAR and 0.36±0.15 logMAR at postoperative 1 and 3 months, respectively (p<0.001). Postoperative mean GCIPL thickness was 66.33±17.28 µm. There was a correlation between mean GCIPL thickness and BCVA at postoperative 3 months (p<0.01). When compared with the control group, GCIPL thickness was significantly thinner in all quadrants of all patients at postoperative 3 months. Dissociated optic nerve fiber layer appearance was observed on C-scan in 13 of 18 eyes postoperatively. There was no correlation between the presence of DONFL and BCVA (p>0.05).

**Conclusion:**

Internal limiting membrane peeling during macular hole surgery may cause functional and/or structural changes that may be associated with visual acuity. Significant GCIPL thinning and DONLF appearance may occur postoperatively.

## INTRODUCTION

The internal limiting membrane (ILM) is the basal lamina of the inner retina and is formed by Müller cells. This basal lamina constitutes the structural interface between the retina and the vitreous humor, and is composed of collagen, glycosaminoglycan, laminins, and fibronectin.^1^ ILM peeling has become a key component of the current vitrectomy technique because it significantly increases the closure rate of macular holes (MH).^2^ In the literature, ILM peeling has been shown to reduce perifoveal traction as well as induce gliosis through surgical trauma, increasing the rate of hole closure.^3^ The rate of MH closure in surgeries performed with ILM peeling has been reported as 90-100%, compared to 60-90% in surgeries without ILM peeling.^1,4,5,6^ In a retrospective study with 18-84 months (mean 44.5 months) of follow-up carried out by Brooks4, functional and visual outcomes of patients with acute and chronic stage II, III, and IV MH were shown to be better among patients who underwent ILM peeling than among those who did not. The authors reported a 100% hole closure rate and a postoperative mean visual acuity of 20/40 in patients who underwent ILM peeling. Studies conducted in Turkey have reported anatomic success rates of 87.5% to 100% after ILM peeling in MH surgery.^7,8,9,10^ However, Haritoglou et al.^11^ showed in their study that more than half of the patients developed paracentral scotomas after ILM peeling. Most of these paracentral scotomas were subclinical, with no change in size, density, and shape reported over time.

In recent years, different spectral domain optical coherence tomography (SD-OCT) devices have been used in several studies to show the effect of ILM peeling on inner retinal layers such as the ganglion cell complex (GCC) after idiopathic MH surgery.^12,13,14^ The GCC has been defined as a region encompassing the nerve fiber layer, the ganglion cell layer, and the inner plexiform layer, and is used to evaluate the morphology of the inner retinal layers.^15^ Baba et al.^13^ reported for the first time that there was thinning of the GCC and subsequent decrease in retinal sensitivity following ILM peeling. Kumagai et al.^16^ demonstrated that there was significant decrease in the GCC in the temporal retina after ILM peeling and that this was associated with a decrease in retinal sensitivity. A new ganglion cell analysis software of the Cirrus HD-OCT (Carl Zeiss Meditec, Dublin, CA, USA) allows measurement of ganglion cell-inner plexiform layer (GCIPL) thickness. This software enables mean and sectoral thickness measurements of the ganglion cell layer, containing ganglion cell bodies, and the inner plexiform layer, containing ganglion cell dendrites.

ILM peeling can cause changes in the inner retinal layers, such as dissociated optic nerve fiber layer (DONFL) appearance.^17,18^ Although DONFL was not previously believed to affect retinal function,^17,19^ another study conducted with microperimetry has shown that retinal sensitivity is reduced in areas with DONFL.^20^

Various dyes are used to make the ILM more visible during surgery.^21,22,23^ Recently, a new dye solution called Membrane Blue-Dual (DORC International, Zuidland, The Netherlands) has come into use. This solution stains the ILM and the epiretinal membrane simultaneously by combining two separate dyes (0.025% Brilliant blue and 0.15% Trypan blue) in the same preparation, thus preventing the need for separate dyes.^24,25^ In addition, the 4% polyethylene glycol component increases the viscosity and density of the dye solution, making the solution heavier and stickier. This eliminates the need for fluid-air exchange.^25^

The aim of the present study was to determine the effect of ILM peeling with the Membrane Blue-Dual dye on GCIPL thickness and the DONFL appearance using the Cirrus HD-OCT device and to investigate the relationship between these changes and visual acuity.

## MATERIALS AND METHODS

The medical records of patients who underwent pars plana vitrectomy with ILM peeling surgery due to idiopathic macular hole were reviewed retrospectively. Age, gender, medical and ocular history details, and presenting complaints were recorded.

Inclusion criteria were:

1. Presence of an idiopathic full-thickness macular hole on SD-OCT,

2. MH closure observed in postoperative OCT images,

3. The absence of a macular hole in the fellow eye, and

4. A follow-up duration of at least 3 months.

Eyes with any other ocular disease which may affect best corrected visual acuity (BCVA), such as glaucoma or uveitis, and eyes that underwent multiple vitrectomies and developed postoperative complications were excluded.

A detailed ophthalmologic examination including slit-lamp biomicroscopy, fundus examination, and intraocular pressure measurement was performed preoperatively on all patients. BCVA was measured using a Snellen chart. Macula images were recorded prior to surgery with fundus photographs, fundus autofluorescence (Heidelberg Retina Angiograph II [HRA2], Heidelberg Engineering, Heidelberg, Germany), and SD-OCT (Cirrus HD-OCT, Carl Zeiss Meditec, Dublin, CA, USA). Patients were evaluated and all measurements were repeated at postoperative 1 day, 1 week, 1 month, and 3 months.

To compare GCIPL thickness at postoperative 3 months, the healthy fellow eyes of the patients and a randomly selected eye of 18 healthy age- and sex-matched individuals were used as a control group. A detailed ophthalmologic examination including slit-lamp biomicroscopy, fundus examination, and intraocular pressure measurement was performed on the healthy control group and the healthy eyes of the patients who had MH surgery. Macular images were recorded with fundus photographs, fundus autofluorescence, and SD-OCT.

OCT measurements were performed with the Cirrus HD-OCT (Carl Zeiss Meditec, Inc., software version 4.0) device after pupil dilation. The base diameter, height, and minimum diameter of the MH were measured manually with OCT. The integrity of the external limiting membrane and inner segment/outer segment (IS/OS) junctional layer was evaluated. The mean and sectoral (superior, inferior, superonasal, superotemporal, inferonasal, inferotemporal) GCIPL thicknesses were measured within the oval ring around the fovea using the macular cube 512x128 protocol with ganglion cell analysis software. Postoperative mean GCIPL thickness was compared with the healthy fellow eyes of the patient and the eyes of the 18 healthy age-matched individuals in the control group. In addition, DONFL presence was evaluated postoperatively in C-scan mode. Measurements were performed preoperatively and at postoperative 1 week, 1 month, and 3 months. To prevent segmentation errors, OCT measurements with a signal strength less than five were not included in the study.

### Surgical Procedure

Sclerotomies were performed with a 23-gauge needle, followed by core vitrectomy. The vitreous humor was stained with intravitreal triamcinolone and the posterior hyaloid was separated. The ILM was stained with intravitreal Membrane Blue-Dual and was peeled using forceps. Fluid-air exchange was done and 20% SF6 was administered. The sclerotomies were not sutured. A sub-Tenon gentamicin-dexamethasone injection was administered. All operations were performed by the same surgeon using the same method.

### Statistical Analysis

SPSS for Windows version 15 software package was used for all statistical analyses. Descriptive statistics were expressed as mean ± standard deviation for variables with normal distribution, as median (minimum-maximum) for variables without normal distribution, and as patient number and percentage for nominal variables.

The significance of intergroup differences in mean values was evaluated using a t-test and the significance of differences in median values was evaluated with the Mann-Whitney U test. The results were considered statistically significant at p values <0.05.

## RESULTS

Eighteen eyes of 18 patients were evaluated. Nine (50%) of the patients were male and 9 (50%) were female. Nine (50%) of the 18 eyes were right eyes and 9 (50%) were left eyes. The mean age of the patients was 65.6±5.6 (55-77) years. Randomly selected 18 eyes of 18 healthy individuals and the patients’ healthy fellow eyes were included in the study as a control group. The demographic data of the patients are shown in Table 1.

**Figure 1:**
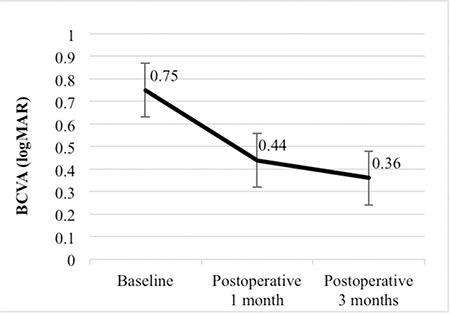
Changes in best corrected visual acuity (BCVA) after macular hole surgery. There is a significant increase in BCVA at postoperative 1 and 3 months (p<0.001, Friedman test) *BCVA: Best corrected visual acuity*

Mean preoperative visual acuity was 0.75±0.19 logMAR; mean visual acuity at postoperative 1 and 3 months was 0.44±0.17 logMAR and 0.36±0.15 logMAR, respectively. The increase in vision at postoperative 1 and 3 months was statistically significant (p<0.001, p<0.001) (Figure 1).

**Figure 2:**
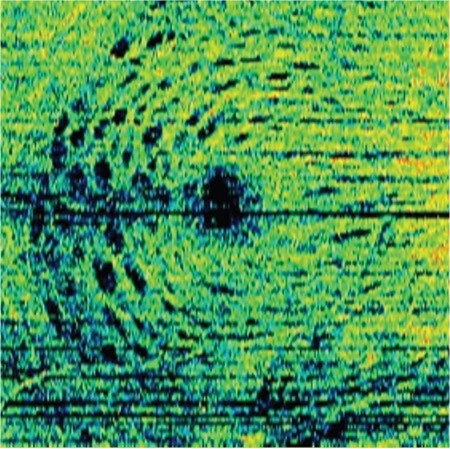
The right eye of a 68-year-old patient; dissociated optic nerve fiber layer appearance is evident in C-scan mode after macular hole surgery

MH base diameter, minimum diameter, and height values were measured manually for all eyes from OCT images prior to surgery. Mean base diameter was 879.16±459.79 (327-1245) μm, minimum diameter was 437.11±238.86 (193-622) μm, and hole height was 454.88±177.45 (348-585) μm. There was no significant relationship between base diameter, hole height, and postoperative BCVA (p>0.05). There was a statistically significant relationship between the minimum MH diameter and BCVA at postoperative 1 month (p=0.026, r=0.522). DONFL was observed on C-scan in 13 of the 18 eyes in the postoperative period (Figure 2). There was no statistically significant relationship between the presence of DONFL and BCVA (p>0.05). A mean of 335.44±143.16 (118-500) μm of IS/OS damage was present in 9 of the 18 eyes. In 12 of the eyes, fundus autofluorescence imaging revealed a hyperfluorescent area with a mean diameter of 496.08±104.64 μm in the region of the hole, but there was no statistically significant relationship between this finding and postoperative BCVA (p=0.466).

We evaluated the GCIPL thickness of the healthy control subjects and MH patients at postoperative 3 months. GCIPL thickness values for all macular sectors (superior, inferior, superonasal, superotemporal, inferonasal, inferotemporal) are shown in Table 2. The mean postoperative 3 month GCIPL thickness of patients who had undergone MH surgery was 52.61±13.97 μm. There was a statistically significant positive correlation between mean GCIPL thickness and postoperative 3 month BCVA (p=0.006, r=0.624). Mean postoperative 3 month GCIPL thickness was significantly thinner in all quadrants in eyes that had undergone MH surgery compared to the eyes of healthy subjects and the patients’ healthy fellow eyes (p<0.001).

There were no intraoperative complications in any of the cases. Four of the 18 eyes underwent cataract surgery at various times after MH surgery.

## DISCUSSION

Studies have indicated that some preoperative parameters such as the duration and diameter of the MH may be associated with postoperative BCVA.^26,27^ In persistent and large MHs, glial cell proliferation causes destruction of the central fovea. ILM peeling in MHs is a controversial topic. Tadayoni et al.^20^ assert that ILM peeling may depend on the diameter of the MH. It has been noted that ILM peeling yields favorable results for MHs larger than 400 μm but does not give the same result for holes with smaller diameters. Ho et al.^28^ reported that total ILM peeling for holes with small diameters may damage the fovea and is not beneficial in terms of visual outcome. In our study, we performed ILM peeling in all cases and found that base diameter did not affect final visual acuity.

The alteration that occurs in the inner retina after ILM peeling has been termed DONFL.^17,18,29,30^ Tadayoni et al.^17^ first reported that arcuate lines extending from the optic nerve to the macula appeared due to ILM peeling in the presence of DONFL. Studies conducted with time domain OCT have shown that DONFL is formed by multiple defects of the nerve fiber layer.18,30 Ito et al.30 reported that the DONFL appeared on OCT as a characteristic focal separation of the optic nerve fiber layer and that functional changes were not seen. The effect of ILM peeling on retinal function is debated in the literature. Two previous studies have shown that there is no significant difference in retinal sensitivity in areas with and without DONFL.^19,31^ However, recent studies have shown that retinal sensitivity may be reduced after ILM peeling, which can be partially explained by SD-OCT images. In these images, it can be seen that the DONFL is not only confined to the nerve fiber layer but also extends to the ganglion cell layer and the inner plexiform layer, thus showing that ILM peeling can lead not only to morphological changes but also to functional changes.^29,32,33,11^ In the present study, a DONFL was observed in the C-scan mode of SD-OCT in 13 of 18 eyes in the postoperative period. There was no relationship between DONFL associated with microtrauma and postoperative BCVA.

Today, agents such as indocyanine green, trypan blue, autologous serum, triamcinolone acetonide, and brilliant blue are used to make the ILM more visible during MH surgery. However, studies have shown that some of these agents may be toxic to retinal neurons and reduce GCC thickness. Among these dyes, brilliant blue seems more reliable because it is cytoprotective towards retinal neurons; however, Baba et al.^34^ demonstrated a reduction in retinal sensitivity and GCC thickness, especially in the temporal quadrant, in MH patients who underwent ILM peeling using brilliant blue. On the other hand, Sevim and Sanisoglu12 showed that the use of brilliant blue did not have an effect on the GCC. In another study involving 32 eyes in which GCIPL thickness was assessed after MH surgery, there was significant thinning in the temporal macular quadrant at postoperative 6 months after ILM peeling with brilliant blue G.^35^ Hashimoto et al.^36^ also reported thinning of the inner retina layers including the ganglion cell and inner plexiform layer, particularly in the parafoveal area, after ILM peeling with brilliant blue G. The authors observed no change in RNFL thickness. In another study including 42 eyes, significant decreases were observed in mean GCIPL thickness and superior sector GCIPL thickness at postoperative 3 and 6 months after ILM peeling with brilliant blue G compared to baseline values, and it was found that this thinning was also accompanied by RNFL thinning.^37^

More recently, the Membrane Blue-Dual dye has been commonly used in MH surgery. In a study of human retinal pigment epithelial cells, electrophysiological evaluations showed that dye applied for up to 5 minutes had no harmful effects on retinal ganglion cells.^38^ In a retrospective comparative case series, successful surgical results were reported with Membrane Blue-Dual dye.^39^ The clinical efficacy of Membrane Blue-Dual dye in macular surgery was compared with ILM blue dye in a prospective, multicenter cohort study including 63 eyes (35 males, 28 females) in the Membrane Blue dye group and 64 eyes (35 males, 29 females) in the ILM blue dye group. With both heavy dye solutions, 80-90% of cases showed postoperative BCVA improvement.^40^

In the present study, ILM peeling was facilitated by Membrane Blue-Dual dye in all patients. GCIPL thickness was found to be significantly thinner in all six sectors of the macular region in surgically treated eyes. In addition, when GCIPL thickness and BCVA at postoperative 1 and 3 months were compared, mean GCIPL thickness was significantly correlated with BCVA at postoperative 3 months. These findings demonstrate that ILM peeling in MH patients may cause both anatomic and functional changes.

The main limitations of the present study are the small number of patients and the short postoperative follow-up period. However, a long-term study showed that the reduction in inner retinal thickness continues until 24 months postoperatively.^16^ Prospective studies with long follow-up periods will be more useful for understanding the morphological changes that occur after vitreoretinal surgery. Another limitation of this study is the absence of a control group comprising eyes treated with vitrectomy without ILM peeling. For this reason, we could not evaluate whether vitrectomy causes direct changes in the ganglion cell layer.

## CONCLUSION

In conclusion, idiopathic MH is a macular pathology that can cause severe vision loss. ILM peeling during MH surgery can cause functional changes and/or structural changes that can be detected with OCT and may be related to visual acuity. Significant GCIPL thinning and DONFL appearance may occur after ILM peeling.

## Figures and Tables

**Table 1:**
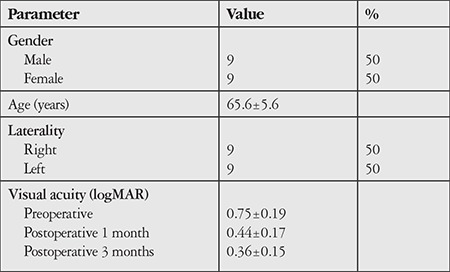
Demographic data of patients with macular hole

**Table 2:**
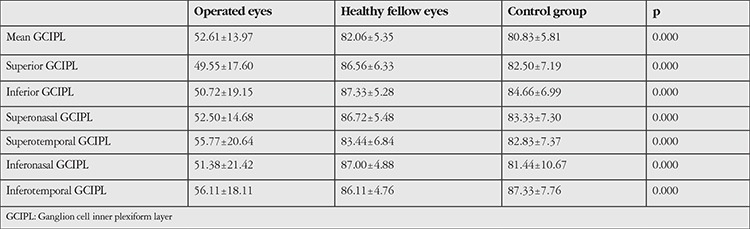
Comparison of average and sectoral ganglion cell inner plexiform layer (GCIPL) thickness (µm) at postoperative 3 months after macular hole surgery in operated eyes, healthy fellow eyes, and the eyes of healthy control subjects
